# Haemophilus influenzae Type f Meningitis With an Intraventricular Abscess

**DOI:** 10.7759/cureus.79187

**Published:** 2025-02-17

**Authors:** Miri Nakashio, Ryoko Nikaido, Junichiro Nishi, Masashi Kasai, Takeshi Morisawa

**Affiliations:** 1 Department of Pediatrics, Kakogawa Central City Hospital, Kakogawa, JPN; 2 Department of Microbiology, Kagoshima University Graduate School of Medical and Dental Sciences, Kagoshima, JPN; 3 Division of Infectious Disease, Hyogo Prefectural Kobe Children's Hospital, Kobe, JPN

**Keywords:** haemophilus influenzae type f, intraventricular abscess, invasive haemophilus influenzae disease, meningitis, β-lactamase-nonproducing ampicillin-resistant

## Abstract

*Haemophilus influenzae* (Hi) is a key pathogen causing bacterial meningitis in children. The introduction of the *Haemophilus influenzae* type b (Hib) vaccine has significantly reduced the incidence of invasive *Haemophilus influenzae* disease (IHD). However, cases caused by non-type b strains, such as type f (*Haemophilus influenzae* type f (Hif)), are gradually increasing, although they remain rare.

We report a case of Hif meningitis complicated by an intraventricular abscess in an 11-month-old boy with no significant medical history. He presented with a high fever and lethargy over two days. Cerebrospinal fluid (CSF) analysis revealed pleocytosis and hypoglycorrhachia, while brain magnetic resonance imaging (MRI) identified an intraventricular abscess. Cultures confirmed the presence of Hif with β-lactamase-negative ampicillin resistance (BLNAR). The patient was treated with intravenous dexamethasone, cefotaxime, and meropenem. Due to a drug rash during cefotaxime therapy, meropenem was reinstated. Follow-up imaging showed complete resolution of the abscess, and the patient recovered fully after a 62-day course of treatment without long-term complications.

This case highlights the emerging threat of invasive Hif infections, particularly BLNAR strains, which complicate treatment options. The report underscores the importance of ongoing surveillance of *Haemophilus influenzae* serotypes and resistance patterns to guide effective management and improve outcomes in such rare but severe infections.

## Introduction

*Haemophilus influenzae* (Hi) is a common resident of the upper respiratory tract and a major pathogen causing infections such as pneumonia and otitis media. Occasionally, it can lead to invasive diseases, including sepsis and meningitis [1]. This bacterium comprises six serotypes (a-f) and non-typeable strains (non-typeable *Haemophilus influenzae* (NTHi)) [1]. Encapsulated strains, compared to NTHi, are more resistant to neutrophil phagocytosis and have a greater tendency for hematogenous dissemination, which contributes to their increased pathogenicity [1]. Among these, type b (*Haemophilus influenzae* type b (Hib)) is the most virulent [1]. Prior to the introduction of the Hib vaccine, Hib was the leading cause of invasive *Haemophilus influenzae* disease (IHD) [1].

The widespread use of the Hib vaccine has significantly reduced the absolute number of IHD cases [1]. As a result, the predominant causative strains of IHD have shifted from type b to non-type b [1]. Among these non-type b strains, NTHi accounts for the majority of cases [1]. Despite this trend, capsular *Haemophilus influenzae*-associated IHD remains rare [1].

Here, we report a case of *Haemophilus influenzae* type f (Hif) meningitis complicated by an intraventricular abscess.

## Case presentation

A previously healthy 11-month-old boy presented to our hospital with a two-day history of high fever and subsequent lethargy. His medical history included short bowel syndrome following neonatal bowel resection for jejunoileal atresia. He had received all scheduled vaccinations, including three doses of the Hib vaccine, which were administered at two, three, and four months of age. Additionally, he had been taking cefditoren pivoxil for two days as prescribed by his previous physician for otitis media. Upon arrival (day 0), the patient exhibited impaired consciousness, with a Glasgow Coma Scale score of 4 (E2V1M1), and pallor. His vital signs were as follows: temperature of 40.8℃, blood pressure of 90/47 mmHg, heart rate of 172 beats per minute, respiratory rate of 44 breaths per minute, and oxygen saturation of 98% (room air). Physical examination revealed no bulging anterior fontanelles or nuchal rigidity. Blood tests (Table 1) showed elevated levels of C-reactive protein (23.2 mg/dL), procalcitonin (26.1 ng/mL), and a white blood cell count of 10,980/μL with neutrophil predominance.

**Table 1:** Laboratory results PaCO_2_: partial pressure of CO_2_, HCO_3_⁻: bicarbonate

Parameter	Result	Normal range
pH	7.4	7.35-7.45
PaCO_2_	34.3	35-45 mmHg
HCO_3_⁻	21.1	22-26 mmol/L
Lactate	2.43	0.5-2.2 mmol/L
Base excess	-3	-2 to +2 mmol/L
White blood cell count	10,980/µL	6,000-17,500/µL
Metamyelocytes	6%	0%
Band neutrophils	8%	0%-5%
Neutrophils	48%	20%-45%
Lymphocytes	35%	40%-70%
Hemoglobin	10.4 g/dL	11.0-13.5 g/dL
Platelet count	226,000/µL	150,000-450,000/µL
Sodium	130 mmol/L	135-145 mmol/L
Potassium	4.1 mmol/L	3.5-5.0 mmol/L
Glucose	93 mg/dL	60-110 mg/dL
C-reactive protein	23.216 mg/dL	<0.5 mg/dL
Procalcitonin	26.13 ng/mL	<0.05 ng/mL
C3	134 mg/dL	65-135 mg/dL
C4	35 mg/dL	15-40 mg/dL
IgG	403 mg/dL	300-900 mg/dL
IgA	36 mg/dL	20-100 mg/dL
IgM	44 mg/dL	30-120 mg/dL

Cerebrospinal fluid (CSF) analysis (Table 2) revealed significant pleocytosis (14,987/μL) and hypoglycorrhachia (glucose level: 1 mg/dL). A cerebrospinal fluid smear suggested the presence of Gram-negative bacilli.

**Table 2:** Cerebrospinal fluid analysis results

Parameter	Result	Normal range
Appearance (color)	Turbid	Clear
Cell count	14,987 cells/µL	0-10 cells/µL
Mononuclear cells	25%	100%
Polymorphonuclear cells	75%	0%
Total protein	312 mg/dL	14-20 mg/dL
Red blood cell count	40 cells/µL	0 cells/µL
Glucose	1 mg/dL	71-90 mg/dL
CSF-to-glucose blood ratio	0.01%	60%-70%

Based on these findings, bacterial meningitis was diagnosed. Treatment with intravenous dexamethasone (0.6 mg/kg/day for two days), cefotaxime (300 mg/kg/day), and meropenem (120 mg/kg/day) was initiated promptly.

On day 2, CSF and blood cultures identified β-lactamase-nonproducing ampicillin-resistant (BLNAR) *Haemophilus influenzae*. Following antimicrobial susceptibility testing (Table 3), therapy was de-escalated to cefotaxime monotherapy.

**Table 3:** MICs and antimicrobial susceptibility testing of Haemophilus influenzae isolated from the patient MICs: minimal inhibitory concentrations, S: susceptible, R: resistant, ST: sulfamethoxazole-trimethoprim

Antimicrobial agent	MIC (µg/mL)	Interpretation
Ampicillin	>4	R
Cefaclor	8	R
Ceftriaxone	0.5	S
Ampicillin/sulbactam	1	R
Imipenem	0.5	S
Meropenem	0.25	S
Azithromycin	1	S
Gatifloxacin	≤0.12	S
Moxifloxacin	≤0.5	S
ST	≤10	S

**Figure 1:**
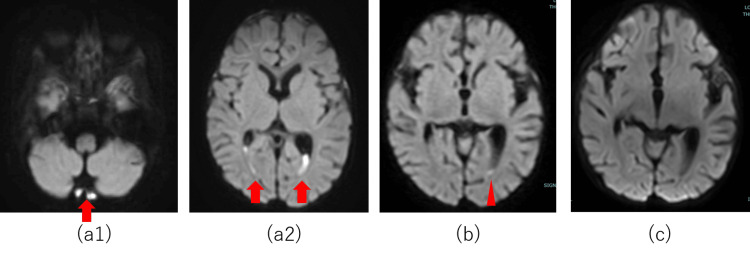
DWI findings on brain MRI On day 5, diffusion-weighted images revealed regions of hyperintensity in the posterior cranial fossa and dorsal horns of the bilateral lateral ventricles (a1 and a2). On day 12, these regions exhibited contractions (b). By day 29, these regions were completely resolved (c). DWI: diffusion-weighted imaging, MRI: magnetic resonance imaging

On day 5, magnetic resonance imaging (MRI) was performed to investigate the persistent fever. Diffusion-weighted imaging of the brain revealed hyperintense areas in the posterior fossa and posterior horns of the bilateral lateral ventricles, suggesting an intracerebral abscess (Figure 1). Given the absence of hydrocephalus and the improvement in blood and CSF test results with antibiotic therapy alone, invasive procedures such as CSF drainage were considered unnecessary. On day 17, a drug rash attributed to cefotaxime necessitated switching to meropenem. A follow-up MRI on day 29 showed complete resolution of the abscess (Figure 1), and by day 55, the CSF cell count decreased (Table 4). The patient completed treatment on day 62 without complications.

**Table 4:** Trends in cerebrospinal fluid analysis results

Hospital day	Day 0	Day 2	Day 5	Day 28	Day 42	Day 55
Cell count	14,987 cells/µL	12,080 cells/µL	232 cells/µL	330 cells/µL	41 cells/µL	19 cells/µL
Total protein	312 mg/dL	190 mg/dL	133 mg/dL	98 mg/dL	56 mg/dL	43 mg/dL
Glucose	1 mg/dL	54 mg/dL	36 mg/dL	34 mg/dL	34 mg/dL	40 mg/dL
CSF-to-glucose blood ratio	0.01%	0.31%	0.34%	0.38%	0.34%	0.38%

Blood examinations performed during hospitalization revealed normal immunoglobulin and complement levels, including normal IgG subclasses. The antibody titer against the Hib vaccine was within the normal range. Lymphocyte subset analysis and lymphocyte proliferation test performed on day 23 of life also showed normal results. Additionally, the Bacillus Calmette-Guérin (BCG) vaccine was administered at five months of age, with no issues observed in the subsequent reaction.

Serotyping identified the isolated *Haemophilus influenzae* as type f, classified as sequence type (ST) 124. PCR analysis confirmed the presence of BLNAR due to mutations in the penicillin-binding protein 3 (*PBP3*) gene.

## Discussion

Based on the present case, we put forth two clinically significant recommendations.

First, the increased virulence of Hif can lead to meningitis. This is partly due to the protein H, found in its capsular membrane and present in most Hib and Hif, which contributes to its strong ability to cause disease [2,3]. Protein H tends to bind with hydronectin, a protein in the blood plasma, which hinders the host's complement system and helps the bacterium adhere to alveolar epithelial cells in the lungs [3]. The binding interaction with hydronectin has been identified in certain surface proteins of NTHi and Hib, suggesting a potential mechanism underlying the high pathogenicity of these bacteria. Additionally, Hif is more genetically stable than other types, and most Hif belongs to a specific genotype called ST124, regardless of regionality [4]. Although several cases of Hif meningitis with intraventricular abscesses have been reported, complete recovery without long-term complications, as observed in this case, is very rare. Sensorineural hearing loss is the most common long-term complication of *Haemophilus influenzae* meningitis. However, in this case, the patient did not develop any complications, likely due to the prompt initiation of appropriate treatment. Hif infections are diverse and severe, often leading to complications. This makes their diagnosis and treatment particularly challenging [4].

Second, it is crucial to consider drug resistance in *Haemophilus influenzae*. While β-lactamase-positive ampicillin-resistant (BLPAR) strains are predominant in the USA and European countries, BLNAR rates have risen to 40% in Japan due to drug misuse, which is particularly concerning [5]. Especially in children under the age of three, BLNAR rates are notably high [6]. This could be due to easy spread among nurseries and peers, frequent antibiotic use, and lower antibody levels against *Haemophilus influenzae*'s common P6 antigen [6]. Many Hif strains are susceptible to penicillin; however, BLNAR Hif strains have also been reported in Japan [7-9]. Following the introduction of public funding for the Hib vaccine in 2011, the first case of Hif meningitis was reported in 2013. Since then, all reported cases of invasive Hif infections were identified as BLPAR strains [7]. However, since 2019, sporadic reports of invasive Hif disease caused by BLNAR strains have emerged, highlighting a concerning trend in antimicrobial resistance [8,9]. This case represents another example of this phenomenon, an infrequent instance of meningitis caused by BLNAR Hif.

Therefore, in Japan, cephem antibiotics should be preferred over penicillin when IHD is suspected. If BLNAR is the causative bacteria, de-escalation can be challenging and the selection of antibacterial agents may be restricted in case of side effects, as observed in this case.

## Conclusions

In conclusion, we report an extremely rare case of Hif meningitis complicated by an intraventricular abscess, highlighting the strong virulence of this capsulated strain.

While non-Hib capsulated strains of *Haemophilus influenzae* have been increasing in Western countries where Hib vaccination was introduced earlier, reports of such cases remain rare in Japan. However, their prevalence may rise in the future. Notably, this case also involved a BLNAR strain, reflecting the high proportion of antibiotic-resistant *Haemophilus influenzae* observed in Japan. This case underscores the necessity of continued monitoring of *Haemophilus influenzae* serotypes and antimicrobial resistance patterns. Systematic data accumulation and surveillance are crucial for tracking trends and implementing effective preventive and therapeutic strategies.
